# Added value of slow diffusion coefficient (SDC) for the separation of benign and malignant parotid gland tumors by diffusion weighted imaging: preliminary results

**DOI:** 10.1186/s12880-025-02051-y

**Published:** 2025-12-01

**Authors:** Dian-Qi Yao, Ann Dorothy King, Rongli Zhang, Ri-Feng Jiang, Lun M. Wong, Yì Xiáng J. Wáng

**Affiliations:** 1 Department of Imaging and Interventional Radiology, Faculty of Medicine, The Chinese University of Hong Kong, Shatin, New Territories, Hong Kong SAR China; 2 Department of Diagnostic Radiology, Li Ka Shing Faculty of Medicine, The University of Hong Kong, Pok Fu Lam, Hong Kong SAR China; 3 Department of Radiology, Fujian Medical University Union Hospital, Fuzhou, Fujian Province China

**Keywords:** Diffusion magnetic resonance imaging, Diffusion derived vessel density, Slow diffusion coefficient, Apparent diffusion coefficient, Parotid gland tumors

## Abstract

**Background:**

Diffusion weighted imaging (DWI) has been used to evaluate parotid gland tumors. Warthin’s tumors (WTs) have very high diffusion-derived vessel ‘density’ (DDVD) and low ADC, while pleomorphic adenomas (PAs) have moderately high DDVD and very high apparent diffusion coefficient (ADC). These two most common benign tumors (BTs) can be largely separated by a combination of ADC and DDVD. However, most of the MTs (malignant tumors) had moderately high DDVD and low ADC, the differentiation between BT and MT remains challenging. Slow diffusion coefficient (SDC) is a novel metric being proposed to measure in vivo tissue slow diffusion. In its basic form, SDC is derived from a high *b*-value DWI image and a higher *b*-value DWI image. This study tested the application of SDC to evaluate parotid gland tumors.

**Methods:**

Twenty-four PAs, 16 WTs, and 14 MTs had DWI at 3T. ADC was calculated with *b* = 0 and 800 s/mm^2^ images. SDC was calculated with *b* = 600 and 800 s/mm^2^ images. DDVD was calculated with *b* = 0 and 20 mm^2^/s images. The ratio of a tumor diffusion metric measure to a contra-lateral tumor-free parotid gland tissue diffusion metric measure was obtained, resulting in ADCr, SDCr, and DDVDr. Pearson test was used for correlation analysis. A receiver operating characteristic curve analysis and the area under the ROC curve (AUROC) were used to assess the diagnostic performance.

**Results:**

Parotid gland tumors had a higher SDC than normal parotid gland tissue with SDCr > 1. SDCr was on average PAs (median: 3.075) > MTs (2.755) > WTs (2.250). Separation of BT against MT by ADCr alone, by a combination of ADCr and SDCr, and by a combination of ADCr, SDCr, and DDVDr had AUROC of 0.7393, 0.8018, 0.8054, respectively. The probability of a tumor being MT is given by: ln(p/1-p) = 0.7006*SDCr-3.198*ADCr + 1.417; or ln(p/1-p)=-0.2225*DDVDr-3.41ADCr + 0.7603*SDCr + 2.103. SDCr was positively correlated with ADCr with a Pearson *r* of 0.624, while SDCr was not apparently correlated with DDVDr.

**Conclusion:**

This study tested the principle of applying four *b*-values DWI to generate three diffusion metrics, namely, ADC, SDC, and DDVD, to evaluate parotid gland tumors. A combination of these three diffusion metrics may offer clinically useful separation of MT from BT.

**Clinical trial number:**

Not applicable.

Among parotid gland tumors (PGT), pleomorphic adenoma (PA) is the most common benign tumor representing 60% of total PGT [1–3], and Warthin’s tumor (WT) is the second most common benign tumor representing 15 to 20% of total PGT [2]. 20 to 30% of PGT are malignant tumor (MT) [4]. The initial diagnosis of a PGT before surgical excision is confirmed by histopathological analysis of specimens obtained by ultrasound-guided fine-needle aspiration or core biopsy. The main role of CT or MRI is to map the location and extent of the tumor prior to surgical resection. When PGT is detected as incidental imaging finding, it is helpful to indicate in the report whether the incidental PGT is likely to be benign or malignant. Imaging can be additionally helpful in characterization in terms of supporting or questioning the provisional cytological diagnosis.

Diffusion weighted imaging (DWI), particularly the diffusion metric apparent diffusion coefficient (ADC), has been used to evaluate PGT. It is well recognized that WTs have a low ADC, while PAs have a high ADC [5–7]. The DWI-derived biomarker DDVD (diffusion-derived ‘vessel density’) works on the principle that on spin-echo type echo-planar DWI images, blood vessels including micro-vessels show high signal when there is no motion probing gradient (*b* = 0 s/mm^2^), while they show low signal even when very low *b*-values (such as *b* = 1–2 s/mm^2^) are applied. Thus, the signal difference between images when the motion probing gradient is ‘off’ and ‘on’ reflects the extent of tissue functional vessel density [8–10]. Recently, DDVD has been tested for the evaluation of PGT tumor perfusion [11]. DDVDr is DDVD of the tumor divided by DDVD of tumor-free parotid gland tissue [11]. It was noted that most of the PGTs were hyper-vascular relative to native parotid gland tissue with DDVDr >1. WT had very high DDVDr, while PA had moderately high DDVDr. A combination of ADC and DDVDr can largely differentiate between PA and WT [11]. However, most of the MTs had moderately high DDVD and lower ADC, the differentiation between BT (benign tumor) and MT remains challenging [11].

Though the diffusion metric ADC has been considered to reflect tissue cellularity, recent work suggests ADC measure is also heavily contributed by tissue T2 relaxation time [12, 13]. To mitigate this, a novel metric slow diffusion coefficient (SDC) has been proposed [14]. SDC was initially tested with its application for the liver and spleen. With the conventional calculation of ADC, the spleen has a much lower ADC than liver, hepatocellular carcinomas (HCCs) have a lower ADC than adjacent liver parenchyma, and simple liver cysts have a higher ADC than liver hemangiomas. On the other hand, SDC analyses suggested that the spleen has a faster diffusion than the liver, HCCs have a faster diffusion than adjacent liver parenchyma, and liver hemangiomas have a faster diffusion than simple liver cysts [14]. The liver and spleen have a similar amount of blood perfusion, the spleen is waterier than the liver, and the spleen tissue has a higher contrast-enhanced CT measured extracellular volume fraction than the liver [15, 16]. As HCCs are mostly associated with increased blood supply and increased proportion of arterial blood supply and with edema, the SDC results appeared to be more reasonable [14]. It is also more reasonable with SDC results that the diffusion of liver hemangioma liquid is faster than the more ‘static’ liquid of the liver cysts. Moreover, abscess liquid has been reported to be of low ADC with diffusion restriction which appears to be unreasonable [17]. SDC measure suggests that liver abscess liquid may have faster diffusion than the adjacent liver parenchyma [18]. In a recent study of 63 patients with diffuse gliomas (30 IDH-mutant and 33 IDH-wildtype), SDC separated the 30 IDH-mutant and 33 IDH-wildtype tumors with receiver operating characteristic area under curve (AUROC) of 0.828. A combination of SDC, DDVD, and ADC separated IDH mutant and IDH-wildtype tumors with an AUROC of 0.897 [19]. The SDC metric may be particularly more reasonable than ADC for tissues of short T2 relaxation time (T2) [14, 20].

The current study tested the application of SDC to evaluate the slow diffusion of PGT, and explored using a combination of ADC, DDVD, and SDC to classify MT against BT. As both PA and WT are BT, the separation between BT and MT will be clinically more relevant than the separation between PA and WT.

## Materials and methods

The study was approved by the local institutional review board with informed consent obtained. Intravoxel incoherent motion (IVIM) data were initially prospectively acquired, and then retrospectively analyzed in the current study. Study subjects included adult patients with parotid gland PA, WT, or MT as confirmed at histopathological examination, and who had undergone MRI with prescribed DWI from June 2013 to March 2021. MRI was performed using a 3T MRI scanner (Achieva TX, Philips Healthcare), with a head and neck coil for radiofrequency transmission and a 16-channel Philips neurovascular phased-array coil for reception. In addition to standard structural MRI, fat-suppressed single-shot spin-echo echo-planar-imaging sequence DWI data were sampled in the axial plane. DWI intravoxel incoherent motion (IVIM) series images were acquired with the following parameters: TR/TE, 2000/50 ms; FOV 256 × 256 mm; 4 mm slice thickness; inter-slice gap: 0.06 mm; voxel size ranged from 2.327 × 2.188 × 4.0 mm to 2.7 × 2.7 × 4.0 mm; number-of-excitation (NEX) = 1. A series of *b*-values of 0, 20, 40, 50, 60, 80, 100, 200, 300, 400, 500, 600, 800, 1000, 1200, 1500 s/mm^2^ were acquired. MRI was conducted on 59 consecutive patients. Tumors were excluded when: (i), tumor was almost entirely cystic/necrotic, with liquid signal on T2 weighted image occupying 2/3 of the lesion area; (ii), tumor contained a large internal hemorrhage occupying 1/2 of the lesion area; or (iii) images with substantial artifacts. The final analysis in this study included 24 PAs, 16 WTs, and 14 MTs (PA and WT are grouped together as BT). There were 16 females and 8 males, with a mean age of 52 years, for the PA group. There were 2 females and 12 males (two male cases have bilateral tumors), with a mean age of 69.4 years, for the WT group. There were 9 females and 5 males, with a mean age of 55.4 years, for the MT group.

**Fig. 1:**
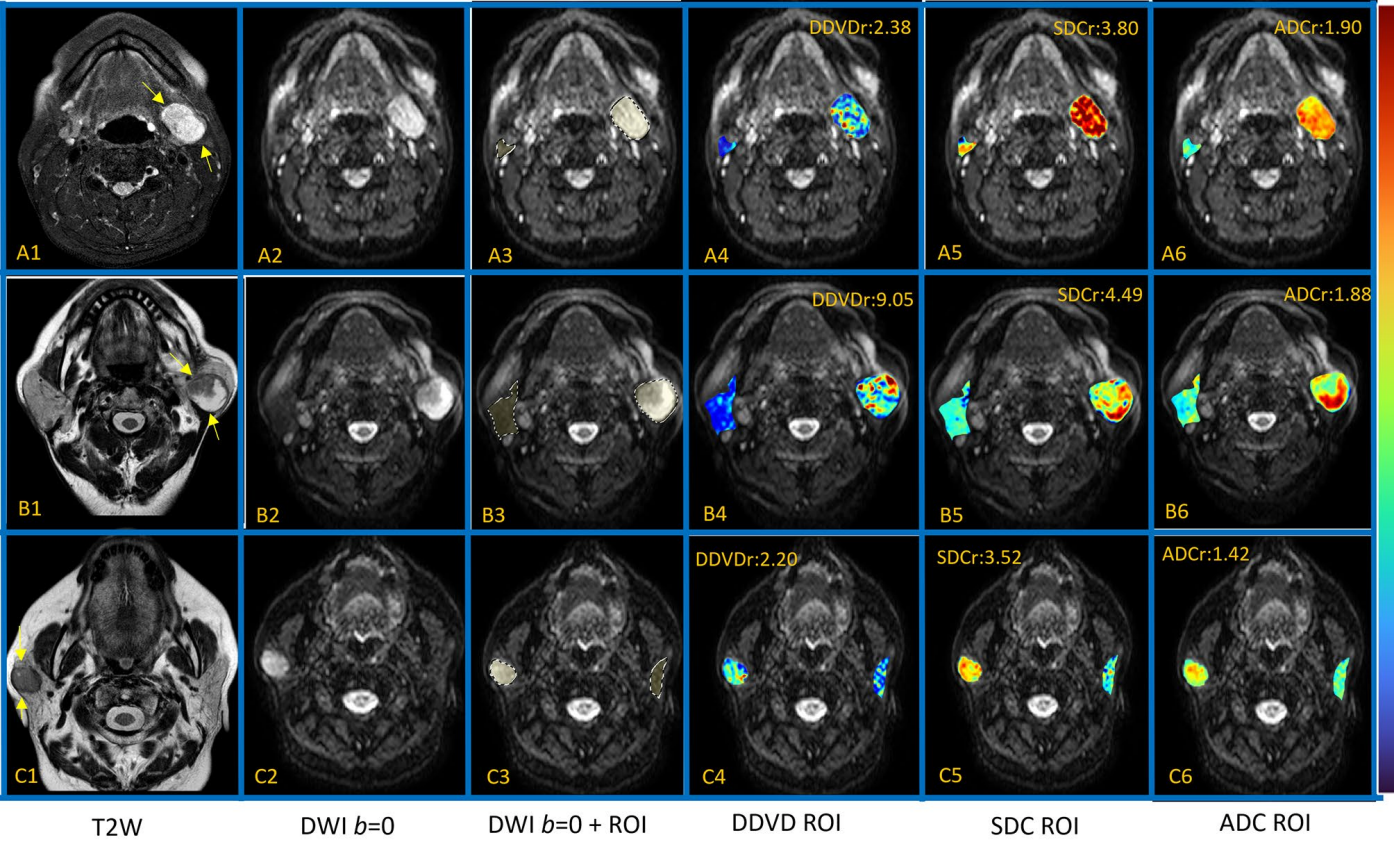
Illustration of three cases of parotid gland tumors. A is a case of PA, B is case of WT, and C is a case of MT. Images include, from the left to the right, T2 weighted image (**A1**-**C1**), *b* = 0 diffusion weighted image (DWI, **A2**-**C2**), *b* = 0 DWI with ROI drawn for the tumor and the contra-lateral parotid gland (**A3**-**C3**), DDVD pixelwise map for the parotid gland ROI and the tumor ROI overlaid on *b* = 0 DWI (**A4**-**C4**), SDC pixelwise map for the parotid gland ROI and the tumor ROI overlaid on *b* = 0 DWI (**A5**-**C5**), ADC pixelwise map for the parotid gland ROI and the tumor ROI overlaid on *b* = 0 DWI (**A6**-**C6**). Values of DDVDr, SDCr, and ADCr are labeled for each tumor

The quantification applied ROI-based analysis. Contours were manually drawn on *b* = 0 s/mm^2^ covering the tumor or contralateral normal parotid gland, and adjusted with reference to anatomical images, excluding necrotic and cystic regions (Fig. 1). Visible vessels near the tumor or parotid gland were also carefully excluded. The ROIs were primarily based on those used in reference 11 (Yao et al.), with further slight tuning to better include the lesion or normal gland. The contours on *b* = 0 s/mm^2^ image were then fitted to the other DW images (images with *b* = 20, 400, 600, 800, 1000 s/mm^2^ utilized in the current study). The contours were then further adjusted on these non-zero b-value images manually. Blinded to the pathological diagnosis of the parotid gland tumors, the contours for ROI analyses were drawn by a trainee radiologist and a trained engineering graduate, then an experienced radiologist checked the quality of the contours and finally consensuses were reached for all ROI segmentations. ADC, SDC, and DDVD were then calculated.

As absolute MR signal intensity is influenced by various factors, including B1 spatial inhomogeneity, coil loading, and receiver gain, etc., in this study the ratio of a tumor diffusion metric measure to a contra-lateral tumor-free parotid gland tissue diffusion metric measure was used to minimize these scaling factors. On diffusion metric pixelwise map, conceptually this approach would be equivalent to a radiologist visually comparing a lesion’s signal to the adjacent tissues’ signal.

ADC was obtained according to:

1 $$ADC = {{\ln (S{b_0}/S{b_{800}})} \over {{b_{800}} - {b_0}}}\,\,\,\,\left[ {unit:m{m^2}/s} \right]$$

where *b*_*800*_ = 800 and *b*_*0*_ = 0 s/mm^2^, *Sb*_*0*_ refers to the signal intensity within the ROI when *b* = 0 s/mm^2^, and *Sb*_*800*_ refers to the signal intensity within the ROI when *b* = 800 s/mm^2^.

2 $$\eqalign{ & {\rm{ADCr = }}\left({{\rm{ROI - based}}\,{\rm{mean}}\,{\rm{ADC}}\,{\rm{of}}\,{\rm{PGT}}} \right) \cr & {\rm{/(ROI - based}}\,{\rm{mean}}\,{\rm{ADC}}\,{\rm{of}}\,{\rm{parotid}}\,{\rm{gland)}} \cr}$$

SDC was obtained according to [14]:

3 $$\eqalign{ {\rm{SDC = }} & \left[ {{\rm{S(}}{{\rm{b}}_{\rm{1}}}{\rm{)-S(}}{{\rm{b}}_{\rm{2}}}{\rm{)}}} \right]{\rm{/(}}{{\rm{b}}_{\rm{2}}}{\rm{-}}{{\rm{b}}_{\rm{1}}}{\rm{)]}} \cr & {\rm{[unit: arbitrary unit (au)/s]}} \cr}$$

4 $$\eqalign{ & {\rm{SDCr = }}\left({{\rm{ROI - based}}\,{\rm{mean}}\,{\rm{SDC}}\,{\rm{of}}\,{\rm{PGT}}} \right) \cr & {\rm{/(ROI - based}}\,{\rm{mean}}\,{\rm{SDC}}\,{\rm{of}}\,{\rm{parotid}}\,{\rm{gland)}} \cr}$$

where *b*_*1*_ and *b*_*2*_ refer to a high *b*-value (e.g., 600 mm^2^/s) and a higher *b*-value respectively (e.g., 800 mm^2^/s), where S(*b*_*1*_) and S(*b*_*2*_) denote the image signal-intensity acquired at the high *b*-value and the higher *b*-value respectively. For comparison, SDC values for additional two pairs of *b-*values of *b*_*1*_ = 400/*b*_*2*_ = 600 and *b*_*1*_ = 800/*b*_*2*_ = 1000 were also obtained.

DDVD was computed using the following Eq. (8):

5 $$\eqalign{ {\rm{DDV}} & {{\rm{D}}_{{\rm{b0b20}}}}{\rm{ = }}{{\rm{S}}_{{\rm{b0}}}}{\rm{/RO}}{{\rm{I}}_{{\rm{area\_b0}}}}{\rm{-}}{{\rm{S}}_{{\rm{b20}}}} \cr & {\rm{/RO}}{{\rm{I}}_{{\rm{area\_b20}}}}\,\,\,\,\left[ {{\rm{unit: au/pixel}}} \right] \cr}$$

Where *S*_*b0*_ and *S*_*b20*_ refer to the sum of signals within the selected ROI on *b* = 0 and 20 s/mm^2^ images, respectively. ROI_area_b0_ and ROI_area_b20_ refer to the ROI area (unit in pixel) on *b* = 0 and 20 s/mm^2^ images.

6 $$\eqalign{ & {\rm{DDVDr = }}\left({{\rm{ROI - based}}\,{\rm{mean}}\,{\rm{DDVD}}\,{\rm{of}}\,{\rm{PGT}}} \right) \cr & {\rm{/(ROI - based}}\,{\rm{mean}}\,{\rm{DDVD}}\,{\rm{of}}\,{\rm{parotid}}\,{\rm{gland)}} \cr}$$

DDVD, ADC, and SDC measures shared the same ROI for each slice when applicable, thus *S*_*b0*_ in Eq. (1) and *S*_*b0*_ in Eq. (5) are the same in this study. The mean of all included slice measurements was regarded as the value of the DWI scan, with the last step weighted by the ROI area of each slice.

In our earlier study with the same image data, for a random selection of 12 cases with a suitable amount of normal parotid gland tissue available bilaterally for analysis, the DDVD values of the left parotid and the right parotid had an intra-reader intraclass correlation coefficient (ICC) of 0.75, and that of the ADC was 0.73 [11]. With the same data, SDCr_b600b800_ for the left parotid and for the right parotid had an intra-reader ICC of 0.783. When 12 tumors were measured by one reader and then the ROIs were edited by another reader, the inter-reader agreement was high with an ICC of 0.997 for SDCr_b600b800_.

Statistical analysis was performed using GraphPad Prism Software (GraphPad Software Inc., San Diego, CA, USA). Comparisons were tested by Kruskal-Wallis test. A receiver operating characteristic curve analysis and the area under the ROC curve (AUROC) were used to assess the diagnostic performance. Pearson test was used for correlation analysis. A P value < 0.05 was considered statistically significant.

## Results

**Fig. 2:**
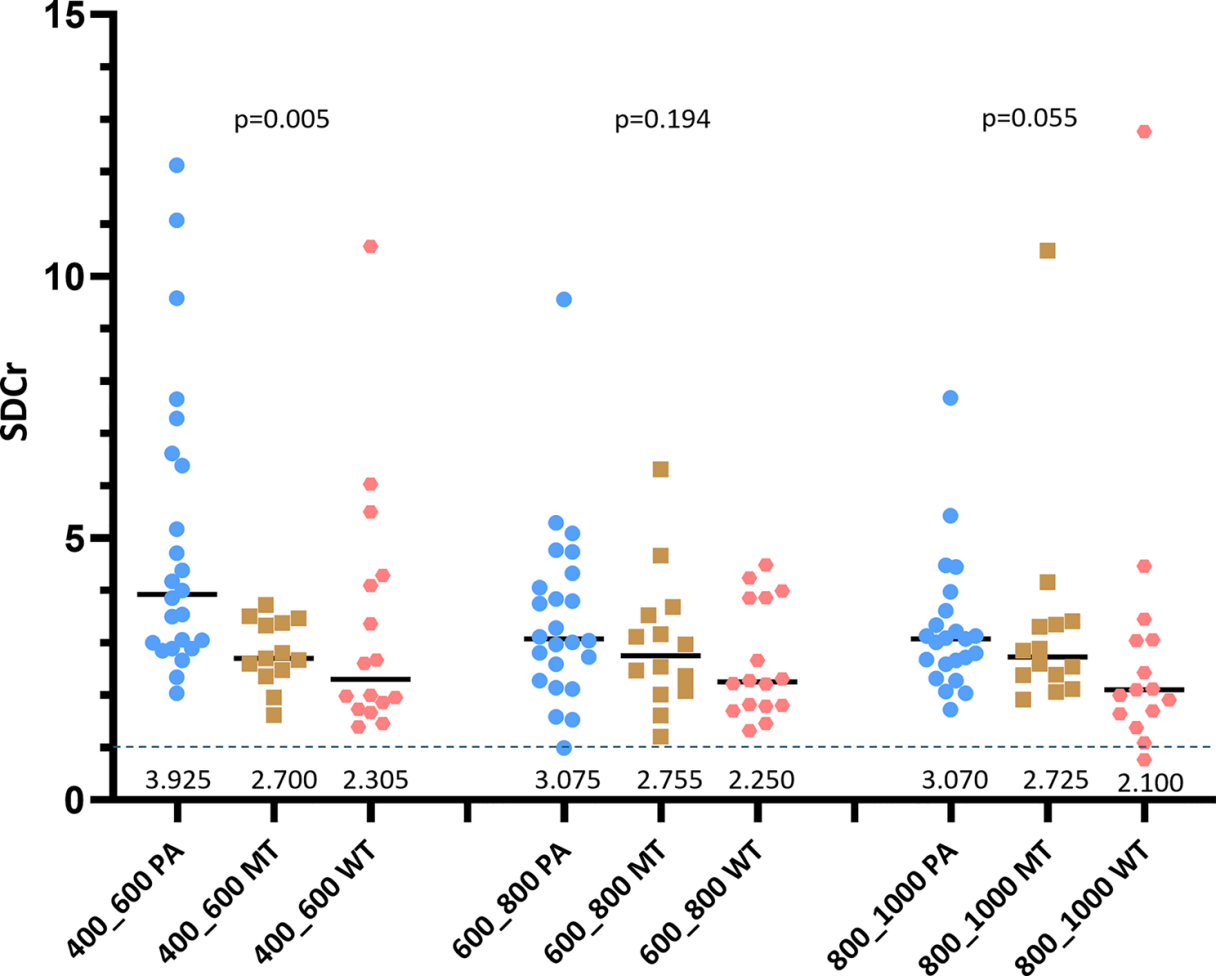
Scatter plot of PA, MT, and WT values. A trend is noted that SDCr values are ‘PA > MT > WT’, regardless of *b*-values = 400 and 600 s/mm^2^, or *b*-values = 600 and 800 s/mm^2^, or *b*-values = 800 and 1000 s/mm^2^, were used to calculate the SDC value. One MT case with image artifact for SDC_b400b600_ calculation was removed from analysis, and one WT case with image artifact for SDC_b800b1000_ calculation was also removed from analysis. Median values are labelled in this graph, *p*-values are based on Kruskal-Wallis test

The SDCr results for PGTs are shown in Table 1*; *Fig. 2. Most of the PGT had a higher SDC than normal parotid gland tissue with SDCr > 1. SDCr was on average PA > MT > WT.

**Fig. 3:**
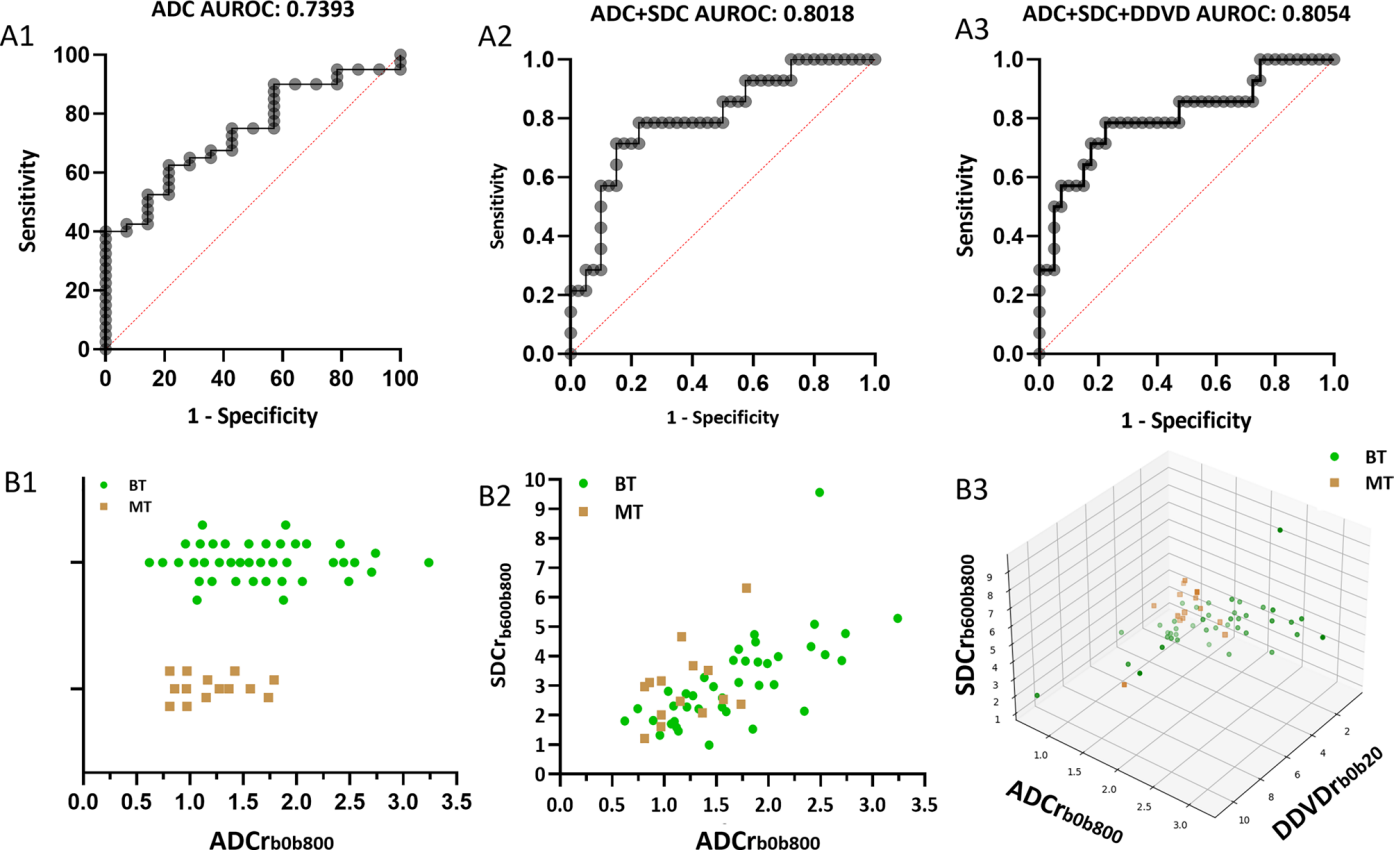
The separation performances for MT against BT by ADCr_b0b800_ alone (A1 and B1), a combination of ADCr_b0b800_ and SDCr_b600b800_ (A2 and B2) and a combination of ADCr_b0b800_, SDCr_b600b800_, and DDVDr_b0b20_ (A3 and B3). A1, A2, and A3: AUROC; B1, B2, and B3: scatter plots

Separation of MT against BT by ADCr alone had AUROC of 0.7393 (Fig. 3A). Separation AUROC of BT against MT by a combination of ADCr and SDCr_b600b800_ improved to 0.8018 (Fig. 3B). Separation AUROC of BT against MT by a combination of ADCr, SDCr_b600b800_, and DDVDr further slightly improved to 0.8054 (Fig. 3C). A combination of ADCr and DDVDr separated MT against BT with an AUROC of 0.7536. For the tumor separation when compared with ADCr and/or DDVDr, AUROC testing showed SDCr_b600b800_ performed slightly better than SDCr_b400b600_ and SDCr_b800b1000_, thus only SDCr_b600b800_ were utilized in the further analyses.

Based on the data shown in Fig. 3A, the probability of a tumor being MT is calculated by:

7 $${\rm{ln}}\left({{\rm{p/1 - p}}} \right){\rm{ = - 1}}{\rm{.872*ADCr + 1}}{\rm{.646}}$$

Based on the data shown in Fig. 3B, the probability of a tumor being MT is calculated by:

8 $${\rm{ln}}\left({{\rm{p/1 - p}}} \right){\rm{ = 0}}{\rm{.7006*SDCr - 3}}{\rm{.198*ADCr + 1}}{\rm{.417}}$$

Based on the data shown in Fig. 3C, the probability of a tumor being MT is calculated by:

9 $$\eqalign{ {\rm{ln}}\left({{\rm{p/1 - p}}} \right) & {\rm{ = - 0}}{\rm{.2225*DDVDr - 3}}{\rm{.41*ADCr}} \cr & {\rm{ + 0}}{\rm{.7603*SDCr + 2}}{\rm{.103}} \cr} $$

**Fig. 4:**
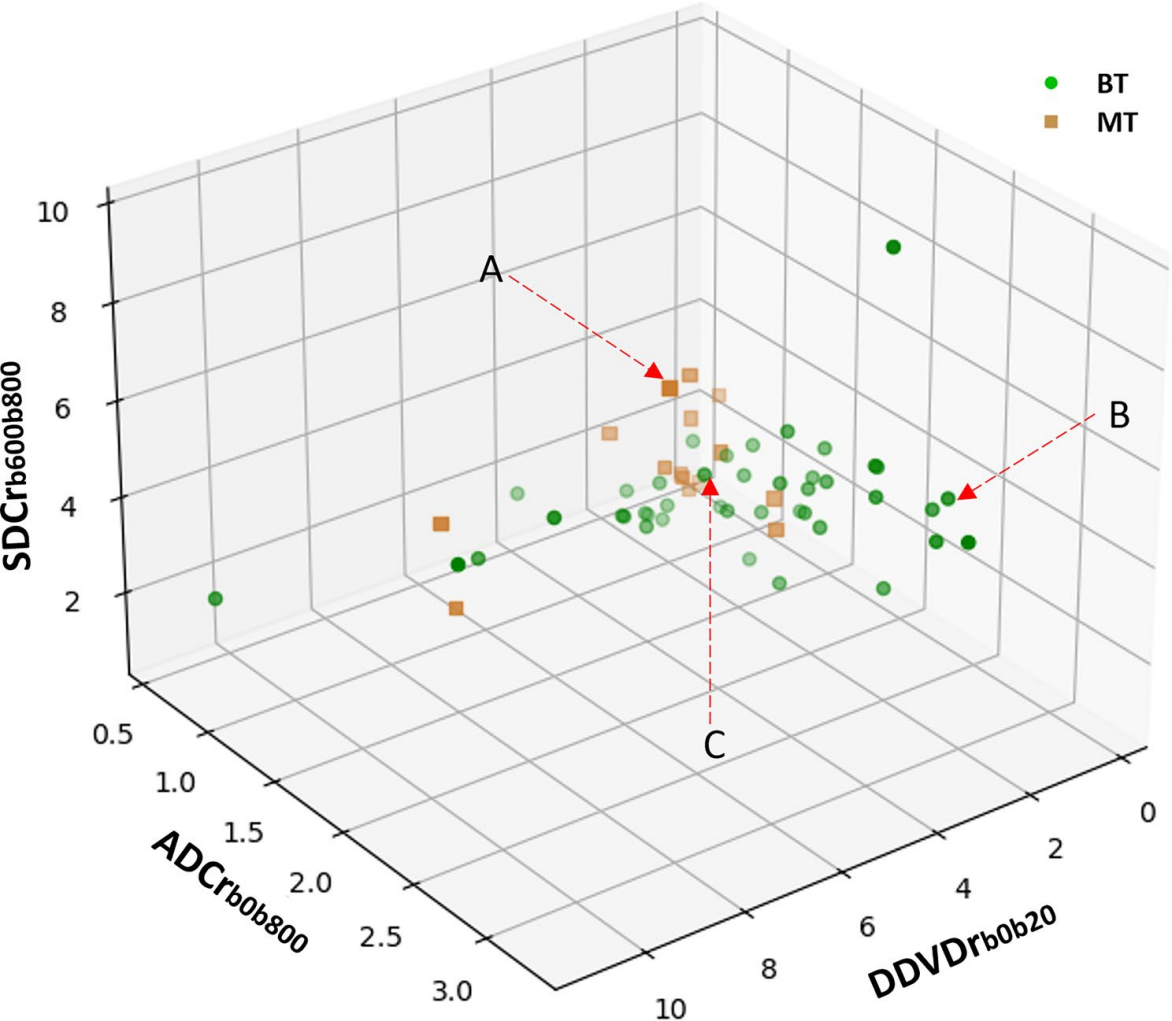
Distribution of patient data in a three-dimensional space with the axes being ADCr_b0b800_, SDCr_b600b800_, and DDVDr_b0b20_. This plot is magnified from Fig. 3B3. It can be visually estimated seen case-A, a mucoepidermoid carcinoma, is likely to be with an MT, case-B, a pleomorphic adenoma, is likely to be with a BT, and case-C, a Warthin’s tumor, is undecidable. Quantitative analysis shows that, case-A had a probability of 0.771 being an MT, case-B had a very low probability of 0.004 being an MT (i.e., very likely to be a BT), case-C had a probability of 0.388 being an MT (i.e., undecidable, but still more likely to be a BT)

Illustration of three cases as denoted Fig. 4 is shown in Table 2. An addition of SDCr to ADCr substantially improved the diagnostic confidence. Consistent with the visualized estimation, case-A was more likely to be an MT (probability = 0.771), case-B was unlikely to be an MT (probability = 0.004), case-C was undecidable (probability = 0.388).

**Fig. 5:**
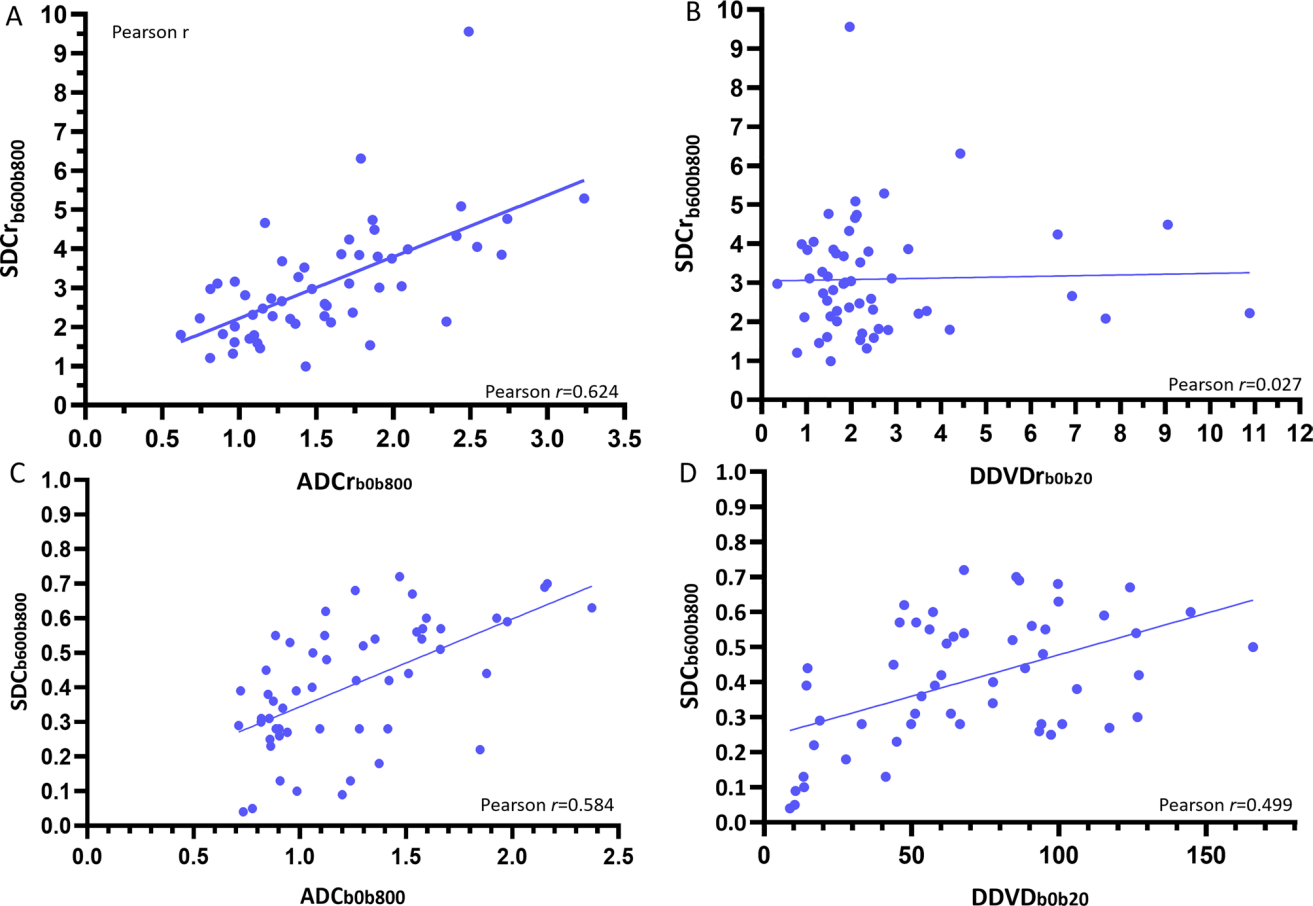
Correlations of SDCr_b600b800_ with ADCr_b0b800_ (**A**) and with DDVDr_b0b20_ (**B**), and correlations of SDC_b600b800_ (unit in a.u./s) with ADC_b0b800_ (unit in × 10^− 3^/mm^2^/s, **C**) with DDVD_b0b20_ (unit in a.u./pixel, **D**). All parotid gland tumors of PA, MT, and WT are grouped together. A positive and modestly strong correlation is noted between SDCr and ADCr (**A**, Pearson *r* = 0.624), while no correlation is noted between DDVDr and ADCr (**B**). A positive and modestly strong correlation is noted between SDC and ADC (**C**, Pearson *r* = 0.584), and between SDC and DDVD (**D**, Pearson *r* = 0.499)

Correlation analysis showed SDCr was positively correlated with ADCr with a Pearson *r* of 0.624, while SDCr was not apparently correlated with DDVDr. A positive and modestly strong correlation was noted between SDC and ADC (Pearson *r* = 0.584), and between SDC and DDVD (Pearson *r* = 0.499, Fig. 5).

## Discussion

This study tested the value of SDC for parotid gland tumors classification. All PGT had a higher SDC than normal parotid gland tissue. SDC value was PA > MTs > WTs. Separation of BT against WT by ADCr alone, by a combination of ADCr and SDCr, and by a combination of ADCr, SDCr, and DDVDr, had AUROC of 0.7393, 0.8018, 0.8054, respectively. Thus, combination of ADC and SDC offers clinically useful separation of MT from BT.

It has been well documented that the groupwise mean of ADC value decreases from PAs to MTs and then to WTs [5–7]. For PA, its high ADC value can be attributed to its myxomatous and chondroid contents which have long T2 relaxation time [12, 13, 21, 22]. For WT, its low ADC can be attributed to mature lymphocytic component that is similar to lymphoid tissue elsewhere in the body [23, 24]. Lymphoid tissues have T2 around 70 ms which demonstrate low ADC measure [13, 25]. With a 3.0T scanner, Baohong et al. [5] reported that T2 was 142.9 ± 53.8 ms for PA, and such long T2 is associated with elevated ADC measure. On the other hand, Baohong et al. reported T2 of 83.27 ± 23.47 ms for WT, and it has been noted that T2 range of 60–80 ms is associated with low ADC [13]. Baohong et al. reported T2 of 97.5 ± 45.2 ms for MT, which is moderately long and thus these tumors are associated with a modest ADC. Histology studies show WTs have abundant tumor vascularity [26–28]. The current study data showed relatively higher DDVDr value and lower ADCr for WT, relatively higher ADCr value and lower DDVDr for PA (Table 1), these are consistent with earlier reports [29–31].

With the same patient data as in this study, we have earlier showed that a combination of DDVDr and ADC could largely separate PA and WT (two types of benign PGTs), however the separation of MT from BT remained more challenging [11]. Other studies with imaging based diffusion and perfusion measures showed similar results [28–31]. In the current study, a pattern was noted with ‘PA SDCr >MT SDCr >WT SDCr’. Statistical significance was achieved for SDCr_b400b600_ measures, marginally achieved for SDCr_b800b1000_ measures, and statistical significance was not achieved for SDCr_b600b800_ measures probably due to the limited sample size and large data distribution range (Fig. 2). Results in the current study show that, separation AUROC of MT against BT improved from 0.7393 by ADCr alone to 0.8018 by a combination of ADCr and SDCr_b600b800_, and further improved to 0.8054 by a combination of ADCr, SDCr_b600b800_, and DDVDr. Moreover, according to our additional testing, a combination of ADCr_b0b1000_, SDCr_b600b800_, and DDVDr derived a slightly further improved AUROC of 0.8143. Our results compared favorably with reported literature results for the separation of parotid gland BT and MT. For example, Ma et al. [31]. reported an AUROC of 0.722 by IVIM-Dslow. Baohong reported an AUROC of 0.705 by T2 value, an AUROC of 0.715 by ADC value, and an AUROC of 0.731 by a combination of T2 value and ADC values [5]. Tao et al. [32]. tested using dynamic contrast-enhanced (DCE) MRI to evaluate PGT. A Gadolinium agent was administered intravenously at a rate of 2mL/s with a total dose of 0.1 mmol/kg body weight. MRI was sequentially obtained prior to and after administration of the contrast material, 40 consecutive data sets were acquired in 180–200 s. The DCE-MRI analysis derived an AUROC of 0.8145 in their study. It is noted that, though DDVDr contributed only slightly for BT and MT classification beyond the combination of ADCr and SDCr, DDVD can be clinically relevant for PGT perfusion assessment and for the separation of PA and WT [11]. DDVDr ratios of MT/PA and WT/PA agreed with the literature perfusion results derived with non-DWI methods, particularly consistent with CT perfusion blood volume results [11].

Nearly all the PGTs had SDCr >1, suggesting that PGTs had faster diffusion than the normal parotid gland tissue. The reasons why PGTs had faster diffusion than parotid gland tissues could not be investigated in this study, but it is likely to be multi-factorial. We have noted that PGTs have DDVDr >1 suggesting higher perfusion for the tumor than for the normal parotid gland tissue, higher tissue perfusion is likely to contribute to a faster diffusion of the tissue. SDCr was positively correlated with ADCr (*r* = 0.624). According to the T2-weighted images in this study, we estimated that 71% of our PAs and 64% of our MTs had higher signal relative to parotid gland tissues, likely reflecting higher free water content and which in turn can be associated with faster tissue diffusion. With the T2 relaxation times of parotid gland tissues and PGTs of MT and PA [5, 33], these tissues are likely to be ‘slow compartment dominant’ according to diffusion MRI model and longer T2 is associated with higher diffusion measures [13]. It did not appear there is a correlation between SDCr and DDVDr for our patients (Fig. 5B). WT has been reported to have a relatively lower ADC measure [5–7]. For WT, only one tumor (i.e., 6%) had higher T2-weighted signal relative to parotid gland tissue in our study. However, WT had much richer perfusion than those of PA and MT, with a DDVDr median value of 3.045 and a mean value of 4.022 in this study (Table 1). Teymoortash et al. [23] found significantly higher intratumoral lymphatic vascular density in WT (mean, 31; SD, ± 4.6) as compared to normal parotid gland (mean, 3; SD, ± 1.1). Similarly, intratumoral blood vessel density was significantly higher in Warthin’s tumor (mean, 81; SD, ± 19.3) than in normal parotid gland (mean, 7; SD, ± 4.2) [23].

There are many limitations to this study. This is a preliminary proof-of-concept study for SDC application in PGT diffusion assessment. We had only 14 patients with MT. MT is heterogeneous while we had very limited sample size for each subtype. We retrospectively analysed our historical GPT IVIM data, the MRI data had only NEX = 1, and the image quality was sub-optimal. The best combination of ADCr, SDCr, and DDVDr for BT and MT separation concerning the *b*-value selection has not been systematically investigated. In this study, the separation AUROC of MT against BT had an AUROC of 0.8054 by a combination of ADCr_b0b800_, SDCr_b600b800_, and DDVDr_b0b20_, while a combination of ADCr_b0b1000_, SDCr_b600b800_, and DDVDr_b0b20_ derived a further improved AUROC of 0.8143. Based on Fig. 2 alone, SDCr_b400b600_ performed better than SDCr_b600b800_ and SDCr_b800b1000_ for the tumor discrimination. However, after we combine SDCr with ADCr and DDVDr, then SDCr_b600b800_ performed slightly better with slightly higher AUROC. This could be due to that the information provided by SDCr_b400b600_ overlapped more with ADCr than the information provided by SDCr_b600b800_. Though this study showed SDCr_b600b800_ performed slightly better than SDCr_b400b600_ and SDCr_b800b1000_, this may further change if the PGT sample composition is different. A lower second *b*-value (such as *b* = 2–10 s/mm^2^, instead of *b* = 20 s/mm^2^ in the current study) for DDVD measure may improve the precision for DDVD value [9, 15]. It is likely that the diagnostic performance of diffusion metrics (i.e., ADC, SDC, DDVD) in PGT characterization will further improve with future technical optimization. This study is focused on the DWI aspects of PGT and particularly with the aim to evaluate the applicability of the novel diffusion metric SDC, it is likely that the integration of PGT morphology into analysis can further help BT and MT separation [32]. Future studies with more optimized DW imaging parameter setups and with larger sample size for various types of tumors are highly desirable.

In conclusion, SDC analysis in this study suggests that the majority of PGT have a faster diffusion measure relative to normal parotid grand tissue. This study tested the principle of applying four *b*-values DWI images to generate three diffusion metrics, namely, ADC, SDC, and DDVD to evaluate PGT. A combination of these three diffusion metrics may offer clinically useful separation of MT from BT, though the precise optimization of probability calculation will require future studies with more optimized DWI parameter setup and with a larger patient data sample size. This four *b*-values DWI approach is likely to be more cost-effective compared with contrast-enhanced imaging and IVIM method which takes longer scan time and often suffers from data fitting instability [11, 26, 34, 35].

**Table 1:** SDCr_b600b800_, ADCr_b0b800_, and DDVDr_b0b20_ values of various parotid gland tumors

Tumor category	Case No.	SDCr (median, mean, 95%CI)	ADCr (median, mean, 95%CI) ^#^	DDVDr (median, mean, 95%CI)^#^
Pleomorphic adenoma	24	3.075, 3.475, 2.590–4.050	1.856, 1.900, 1.553–2.344	1.750, 1.769, 1.490–2.090
Warthin’s tumor’	16	2.250, 2.625, 1.790–3.860	1.175, 1.342, 0.9577–1.713	3.045, 4.022, 2.240–6.600
Malignant tumors	14	2.755, 2.979, 2.010–3.680	1.159, 1.205, 0.8580–1.567	1.890, 2.318, 1.460–2.900
Lymphoepithelioma-like carcinoma	2	1.840, 1.840, 1.210–2.470	0.9818, 0.9818, 0.8108–1.153	1.485, 1.485, 0.7900–2.180
Metastatic carcinoma	2	3.395, 3.395, 3.110–3.680	1.067, 1.067, 0.8580–1.277	2.365, 2.365, 1.830–2.900
Adenoid cystic carcinoma	1	2.966	0.8113	0.3500
Epithelial myoepithelial carcinoma	1	1.610	0.9681	1.460
Mucoepidermoid carcinoma	6	2.850, 3.043, 2.010–4.660	1.294, 1.305, 0.9712–1.736	1.815, 1.807, 1.460–2.200
Acinic cell carcinoma	1	6.310	1.789	4.430
Basal cell adenocarcinoma	1	2.080	1.365	7.670

**Table 2:** The calculation of the probability of being MT for three cases illustrated in Fig. 4

Diffusion metric	Cases	ADCr	SDCr	DDVDr	probability for being MT
ADCr only	A	1.17			0.367
	B	2.34			0.013
	C	1.09			0.402
ADCr + SDCr	A	1.17	4.66		0.720
	B	2.34	2.14		0.010
	C	1.09	2.31		0.389
ADCr + SDCr + DDVDr	A	1.17	4.66	2.08	0.771
	B	2.34	2.14	1.53	0.004
	C	1.09	2.31	2.48	0.388

SDCr_b600b800_, ADCr_b0b800_, and DDVDr_b0b20_ values were used for the calculation. A was a case of MT (mucoepidermoid carcinoma), B was a case of pleomorphic adenoma, and C was a case of warthin’s tumor

## Data Availability

The datasets used and analysed during the current study are available from the corresponding author upon reasonable request.

## Abbreviations

ADC: Apparent diffusion coefficient
ADCr: Apparent diffusion coefficient ratio
BT: Benign tumor
DCE: Dynamic contrast-enhanced
DDVD: Diffusion derived vessel density
DDVDr: Diffusion derived vessel density ratio
DWI: Diffusion-weighted imaging
SDC: Slow diffusion coefficient
SDCr: Slow diffusion coefficient ratio
HCC: Hepatocellular carcinomas
IVIM: Intravoxel incoherent motion
MT: Malignant tumor
PA: Pleomorphic adenoma
PGT: Parotid gland tumor
WT: Warthin’s tumor
AUROC: Receiver operating characteristic area under curve
ROI: Region of interest
